# Oleodaphnoic Acid and Coriaceol, Two New Natural Products from the Stem Bark of *Wikstroemia coriacea*

**DOI:** 10.3390/molecules18032988

**Published:** 2013-03-05

**Authors:** Nicolas Ingert, Isabelle Bombarda, Gaëtan Herbette, Robert Faure, Christian Moretti, Phila Raharivelomanana

**Affiliations:** 1 EIMS, UMR 241 Ecosystèmes Insulaires Océaniens Université de la Polynésie Française, Tahiti 98702 FAA’A, French Polynesia; 2 Centre Polynésien de Recherche sur la Biodiversité Insulaire, UMR 7138, IRD, Papeete BP 529-98713, French Polynesia; 3 LISA EA 4672, Aix-Marseille Université, Campus de St Jérôme, Marseille 13397, cedex 20, France; 4 Spectropole, FR1739, Aix-Marseille Université, Campus de St Jérôme - service 511, Marseille 13397, cedex 20, France; 5 SMBSO, UMR 7273, ICR, Aix-Marseille Université, Campus de St Jérôme - service 522, Marseille 13397, cedex 20, France

**Keywords:** oleodaphnoic acid, coriaceol, *Wikstroemia coriaceae*, Thymelaeaceae, guaiane, 1,5-diphenyl-1-pentanone

## Abstract

Fractionation of the chloroform extract of *Wikstroemia coriacea* led to the isolation of two new compounds, oleodaphnoic acid (**1**), a guaiane-type sesquiterpenoid, and coriaceol (**2**), an 1,5-diphenyl-1-pentanone analogue, together with nine known compounds. The structures of **1** and **2** were elucidated by extensive spectroscopic data analysis. The known compounds were oleodaphnal (**3**), indicanone (**4**), (5*R*,8*R*,8a*R*)-3,8-dimethyl-4,5,6,7,8,8a-hexahydro-5-(1-methylethenyl)-2(1*H*)-azulenone, (**5**), 1,5 diphenyl-1-pentanone (**6**), (+)-3-hydroxy-1,5-diphenyl-1-pentanone (**7**), umbelliferone (**8**), daphnoretin (**9**), β-sitostenone (**10**) and (−)-hinokinin (**11**).

## 1. Introduction

**Figure 1:**
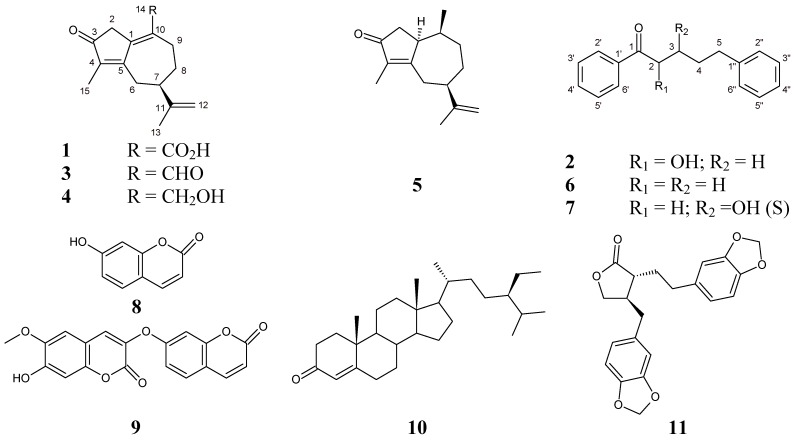
Chemical structures of compounds **1**–**11**.

*Wikstroemia* (Thymelaeaceae) is a genus consisting of about 70 species indigenous to Asia, Malaysia, Australia and Pacific Islands [1]. *Wikstroemia coriacea* B.C. Seemann [2] is an endemic plant, widely distributed in Eastern Polynesia, which is used in folk medicine for its emetic, purgative, narcotic and vesicant properties [3]. Moreover, leaves and stems were used for the treatment of syphilis, gonorrhea, urethritis and leucorrhea [4]. Although there are many phytochemical reports on the genus *Wikstroemia* focused on sesquiterpenes [5,6], diterpenes [7,8,9,10], triterpenes [7,11], coumarins [12,13,14], flavonoids [15,16,17] and lignans [7,18,19,20], no study has been carried out on the chemistry of *Wikstroemia coriacea*. This paper reports our phytochemical discovery of two new natural compounds, oleodaphnoic acid (**1**), a guaiane-type sesquiterpenoid, and coriaceol (**2**), an 1,5-diphenyl-1-pentanone (**6**) analogue, along with nine known compounds including oleodaphnal (**3**), indicanone (**4**), (5*R*,8R,8aR)-3,8-dimethyl-4,5,6,7,8,8a-hexahydro-5-(1-methylethenyl)-2(1*H*)-azulenone, (**5**), 1,5-diphenyl-1-pentanone (**6**), (+)-3-hydroxy-1,5-diphenyl-1-pentanone (**7**), umbelliferone (**8**), daphnoretin (**9**), β-sitostenone (**10**) and (−)-hinokinin (**11**) (Figure 1). In addition, (−)-hinokinin (**11**) was isolated for the first time in the genus *Wikstroemia*.

## 2. Results and Discussion

**Figure 2:**
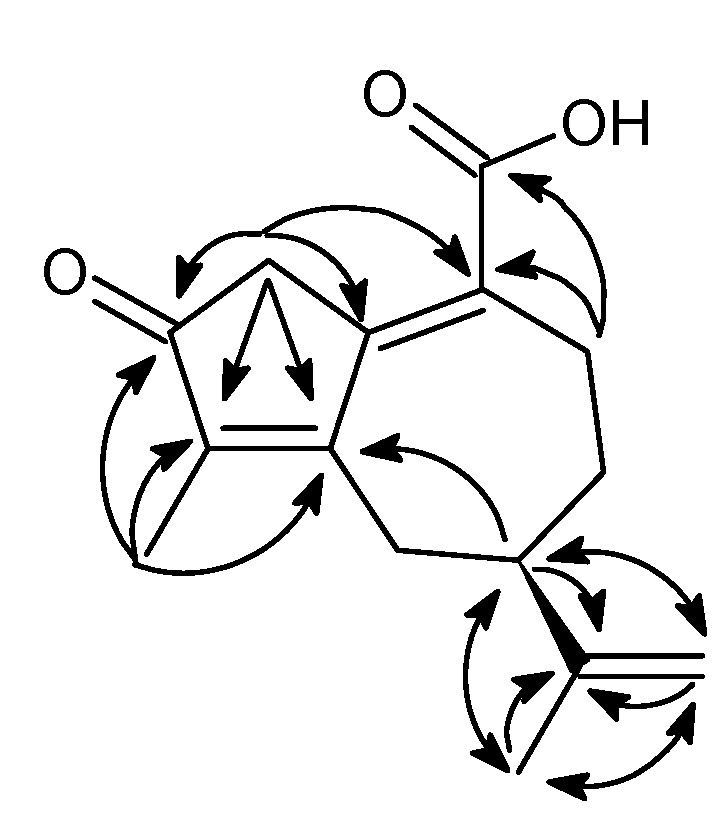
Key HMBC Correlations (H→C) of oleodaphnoic acid (**1**).

A total of eleven compounds were identified from the stem bark of *W. coriacea*. A new sesquiterpenoid oleodaphnoic acid (**1**, Figure 1) was isolated as a colorless powder. Its molecular formula C_15_H_18_O_3_ was assigned on the basis of HR-ESI-MS (*m/z* 247.1326 [M+H]^+^, calcd. 247.1329), implying seven degrees of unsaturation. The IR spectrum displayed significant bands for an unsaturated ketone (1681 cm^−1^) and an α,β,γ,δ unsaturated carboxylic acid (3373 and 1646 cm^−1^). The ^1^H-NMR spectrum showed two tertiary methyl groups as singlets (δ 1.85 and 1.77), and an exo-methylene resonance as two broad singlets (δ 4.76 and 4.75) (Table 1). The ^13^C-DEPTQ NMR spectrum exhibited 15 carbon resonances, including two methyls, four methylenes, one *exo*-methylene, one ketone, one carboxylic function and two double bonds (Table 1). From the COSY and HSQC spectra, the occurrence of both partial structures, CH_3_C=CH_2_ and CH_2_CH_2_CHCH_2_, was suggested. In the HMBC diagram, cross-peak observed between the methyl protons H-13 and the methine carbon C-7 was indicative of the combination of the above partial structures. Further analysis of the other significant long-range ^1^H-^13^C correlations (Figure 2) suggested that NMR data were typical of a guaiane-type skeleton [21]. NMR data close similarity regarding the C-7 carbons of oleodaphnal (**3**) [22], indicanone (**4**) [6] and (5*R*,8*R*,8a*R*)-3,8-dimethyl-4,5,6,7,8,8a-hexahydro-5-(1-methylethenyl)-2(1*H*)-azulenone (**5**) [23], respectively, allowed to infer the *R* stereochemistry of C-7. Finally, the carboxylic group was assigned to be at the C-10 position based on the HMBC relationship between H-9 and C-14. From the above results the structure of oleodaphnoic acid was formulated as **1**.

**Table 1:** ^1^H-NMR (500 MHz,) and ^13^C-NMR (125 MHz,) data of oleodaphnoic acid (**1**) (CDCl_3_) and coriaceol (**2**) (CD_3_OD).

Position	1		Position	2	
	^1^H (δ)	^13^C (δ)		^1^H (δ)	^13^C (δ)
1	-	149.5	1	-	203.2
2	3.38 (brs, 2H)	42.2	2	5.10 (dd, 8.4; 3.3, 1H)	74.1
3	-	204.6	3	1.83 (m, 1H)	35.4
4	-	144.7		1.59 (m, 1H)	
5	-	166.1	4	1.83 (m, 1H)	27.9
6	2.81 (m, 2H)	32.1		1.74 (m, 1H)	
7	2.54 (quint, 7.3, 1H)	42.5	5	2.65 (dt, 14.0; 7.3, 1H)	36.3
8	2.01 (m, 1H)	32.0		2.60 (dt, 14.0; 7.3, 1H)	
	1.81 (m, 1H)		1'	-	136.0
9	2.80 (m, 2H)	25.6	2', 6'	7.90 (m, 2H)	129.5
10	-	127.7	3', 5'	7.48 (m, 2H)	129.9
11	-	148.7	4'	7.61 (m, 1H)	134.6
12	4.76 (brs, 1H)	110.1	1''	-	143.2
	4.75 (brs, 1H)		2'', 6''	7.13 (m, 2H)	129.3
13	1.77 (s, 3H)	20.9	3'', 5''	7.22 (m, 2H)	129.4
14	-	172.7	4''	7.13 (m, 1H)	126.8
15	1.85 (s, 3H)	9.0			

A new natural phenylphenalenone named coriaceol (**2**, Figure 1) was obtained as an yellowish oil and showed an accurate [M+H]^+^ ion at *m/z* 255.1381 (calcd. 255,1379) in the HR-ESI-MS corresponding to the empirical molecular formula C_17_H_18_O_2_ and implying nine degrees of unsaturation. The IR spectrum of **2** suggested the presence of hydroxyl group (3440 cm^−1^), conjugated ketone carbonyl (1683 cm^−1^) and aromatic rings (1603–1480 cm^−1^). The ^13^C-NMR spectrum gave a total of 17 separate resonances and the ^13^C-DEPTQ sequence showed three methylene, eleven methine groups and three quaternary carbons, including a conjugated ketone carbonyl at δ 203.2 (Table 1). The ^1^H-NMR spectrum exhibited two AA’MM’X spin systems, typical of two monosubstituted aromatic rings and an oxygen-bearing methine signal at δ 5.10 (dd, *J* = 8.4, 3.3 Hz) (Table 1). With the aid of COSY experiments, a -OCH(CH_2_)_3_ subunit was identified by further analysis of the remaining ^1^H resonances. Finally, the location of the two phenyl moieties was supported by the HMBC correlations observed between the *ortho* H-2' (d 7.90) and H-2'' (d 7.13) signals and carbons C-1 and CH_2_-5, respectively. From the above spectral data, the structure of coriaceol (**2**) was established as 2-hydroxy-1,5-diphenylpentan-1-one. This compound, which was previously reported with no NMR data as a synthetic intermediate [24], had not yet previously isolated from a natural source. Attempt to determine the stereochemistry at C-2 was not successful due to the decomposition of the compound.

In addition to these two new structures, we isolated three sesquiterpenoids: oleodaphnal (**3**) [22], indicanone (**4**) [6], (5*R*,8*R*,8a*R*)-3,8-dimethyl-4,5,6,7,8,8a-hexahydro-5-(1-methylethenyl)-2(1*H*)-azulenone, (**5**) [23]; two phenylphenalenones: 1,5-diphenyl-1-pentanone (**6**) [25], (+)-3-hydroxy-1,5-diphenyl-1-pentanone (**7**) [26]; two coumarins: umbelliferone (**8**) [27], daphnoretin (**9**) [28]; one triterpenoid: β-sitostenone (**10**) [29] and one lignan: (−)-hinokinin (**11**) [30] (Figure 1). The identifications of these nine known compounds were confirmed by comparison of their physical and spectroscopic data (UV, ^1^H, ^13^C-NMR, MS and [α]) with the corresponding authentic samples or with values described in the literature.

Literature data reported that most of the isolated compounds are known to possess interesting biological activities such as: indicanone (**4**) for its anti-inflammatory activity [6]; (5R,8R,8aR)-3,8-dimethyl-4,5,6,7,8,8a-hexahydro-5-(1-methylethenyl)-2(1*H*)-azulenone (**5**) for its cytotoxic activity on the P-388 cell line [5,31]; (+)-3-hydroxy-1,5-diphenyl-1-pentanone (**7**) for its anti-HIV activity [26]; umbelliferone (**8**) for its anti-inflammatory activities [27], antioxidant [32,33], antihyperlipidemic [34] and anticancer [35,36,37], daphnoretin (**9**) for its antifungal [38], anticancer [15], inhibition of various sites in DNA synthesized voice [14], activation of protein kinase C (platelet aggregation) [39,40], antiviral hepatitis B [41] and respiratory syncytial virus (RSV) properties [42]; the triterpenoid β-sitostenone (**10**) for its interesting biological activities as a strong hypoglycemic [43] and antiarrhythmic [44,45], anti-emetic [46], vasodilator agent [47], and also for its antituberculosis activity [45,48] and anti-inflammatory activity [49]; (−)-hinokinine for its antiparasitic, antifungal [50], antigenotoxic and antioxidant activities [30].

## 3. Experimental

### 3.1. General

HPLC was performed using an Agilent 1100 pump equipped with a Varian Dynamax Microsorb Si column (250 × 21.4 mm i.d., 5 μm, 100 Å) and a Varian Dynamax Microsorb C18 column (250 × 10 mm i.d., 5 μm, 100 Å), respectively, a refractomeric and a Diode Array Detector (DAD) detector. Optical rotations were measured on a Perkin-Elmer model 241 polarimeter equipped with a sodium lamp (589 nm) and a 1 dm cell. HRMS experiments were performed with a QStar Elite mass spectrometer (Applied Biosystems SCIEX) equipped with an ESI source operated in the positive ion mode. IR spectra were obtained with cell composed of two calcium fluoride windows separated by 0.21-mm thick PTFE spacer A145 using a Bruker FTIR Vertex 70 spectrometer. NMR spectra were recorded at 300 K for ~1 mg samples using a Bruker Avance DRX 500 spectrometer, equipped with a Bruker Cryoplateform and a 5 mm TXI cryoprobe. NMR spectra were referenced to CDCl_3_ (δ_H_ = 7.26 ppm and δ_C_ = 77.16 ppm) or to CD_3_OD (δ_H_ = 3.31 ppm and δ_C_ = 49.00 ppm) [51]. Standard Bruker pulse sequences were used for homonuclear and heteronuclear two-dimensional experiments.

### 3.2. Plant Material

The stem bark of *W. coriacea* was collected from Nuku Hiva, Marquesas Islands, and was identified by Dr Jean-François Butaud. A voucher specimen (CM 1725) has been deposited in the Herbarium of French Polynesia [52].

### 3.3. Extraction and Isolation

Air-dried and powdered stem bark of *W. coriacea* (150 g) was extracted with chloroform (3 × 350 mL, 3h rt.) for 10 h. After concentration *in vacuo*, the remaining solid (7.75 g) was subjected to low pressure chromatography (SiO_2_; CHCl_3_/MeOH, 1:0 to 9:1, v:v, then MeOH) to yield three fractions F_1_ (3.2 g), F_2_ (2.8 g) and F_3_ (1.5 g). F_1_ (79 mg) was chromatographed by semi-preparative HPLC (SiO_2_; hexane/isopropanol, 99:1, v:v, 10 mL/min) to provide oleodaphnal (**3**, 4.4 mg), (5R,8R,8aR)-3,8-dimethyl-4,5,6,7,8,8a-hexahydro-5-(1-methylethenyl)-2(1*H*)-azulenone, (**5**, 4.5 mg), 1,5-diphenyl-1-pentanone (**6**, 4.6 mg), 2-hydroxy-1,5-diphenyl-1-pentanone (**2**, 2.8 mg), and (+)-3-hydroxy-1,5-diphenyl-1-pentanone (**7**, 1.9 mg), β-sitostenone (**10**, 1 mg) and (−)-hinokinin (**11**, 0.6 mg). F_2_ (1.34 g) was fractioned using LH20 (CH_2_Cl_2_/MeOH, 1:1, v:v) to isolate umbelliferone (**8**, 68 mg), daphnoretin (**9**, 40 mg) and indicanone (**4**, 7.7 mg). Oleodaphnoic acid (**1**, 11 mg) was obtained from F_3_ (108 mg) through semi-preparative HPLC (C18; H_2_O /EtOH, 7:3 to 0:1 for 30 min then 0:1 10 min, v:v, 2.3 mL/min).

*Oleodaphnoic acid* (**1**). Colorless powder; C_15_H_18_O_3_; 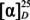 +8 (c 0.02 CHCl_3_); HR-ESI-MS *m/z* 247.1326 [M+H]^+^, calcd. 247.1329; FTIR (CCl_4_) ν_max_ 3373, 1681, 1646, 1599, 1516, 1448, 1384 cm^−1^; ^1^H- and ^13^C-NMR (CDCl_3_) data, see Table 1.

*Coriaceol* (**2**). Yellowish oil; C_17_H_18_O_2_; 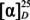 −12 (c 0.003 MeOH); HR-ESI-MS *m/z* 255.1381 [M+H]^+^, calcd. 255.1379; FTIR (CCl_4_) ν_max_ 3440, 1683, 1646, 1593, 1578, 1490, 1362 cm^−1^; ^1^H- and ^13^C-NMR (CD_3_OD) data, see Table 1.

## 4. Conclusions

This work is part of our ongoing phytochemical studies on Polynesian endemic plants aiming at a better knowledge of Polynesian plant biodiversity. We report herein the first phytochemical assessment of the stem bark of *W. coriacea* with the occurrence of two new natural compounds oleodaphnoic acid (**1**) and and coriaceol (**2**), a 1,5-diphenyl-1-pentanone analogue, beside nine known ones. The identified components belong to different secondary metabolite classes including guaiane-type sesquiterpenoids (compounds **1**, **3**, **4**, **5**), triterpenoids (**10**), phenylphenalenones (**2**, **6**, **7**), coumarins (**8**, **9**) and a lignan (**11**) which raises questions about the complexity of the biosynthetic routes to yield such less common chemodiversity exhibited by the same plant. Most of the isolated compounds are known for their relevant biological activities, which add more interest to this endemic Polynesian plant. We will follow up phytochemical analysis along with phylogenetic studies of all endemic species belonging to *Wikstroemia* genus grown in Polynesia aiming at a biodiversity assessment regarding insular plant adaptation and evolution.
